# Catgut Sutures in Surgical Operations

**Published:** 1878-07

**Authors:** 


### MISCELLANEOUS.

### Catgut Sutures in Surgical Operations.

The *Archives de Tocologie des Maladies des Femmes*, a leading Paris monthly, devoted, as its name indicates, to Obstetrics and Diseases of Women, quotes very freely from American medical journals, but rarely has an American contributor. We observe that the number for February contains an original article, (not a translation from English,) from the pen of one of our friends and contributors, Prof. Edward W. Jenks, of Detroit, entitled, "*Sur les sutures de l' uterus dans l' operation Cesarienne*," (Upon Sutures of the Uterus in the Cesarean Operation.)

There are some points in the paper which we deem of importance as bearing upon the Cesarian operation, or any other operation within the peritoneal covity, and the value of catgut as a reliable material for sutures, and we consequently give a brief summary of it.

The *Archives de Tocologie* had translated in full the paper of Dr. Jenks, which also appeared in the journal for December, 1877, upon a successful case of Cesarian section after seven days' labor, in which the writer spoke favorably of catgut sutures for closing the uterine rent as peferable to silver or any other material, although in the case related silver had been used, as catgut was not attainable. Dr. Jenks, for reasons which he gives, having changed his opinion concerning catgut, asks that the same publicity be given to his reason of repudiating catgut, as given to the paper which commended that material. He says that theoretically it is the best material on account of its innocuity and its ready absorption, but practically he believes it the worst of any, as no one yet has devised a knot which the heat and moisture of the peritoneal cavity will not cause to relax and it then be rendered useless. He has employed catgut to ligate intraperitoneal vessels in an ovariotomy where post mortem examination revealed not only the knot untied, but that it was less innocuous than silk used at the same time. In plastic operations about the vagina, a fair test of the same material has convinced him of its utter unreliability. As the only object of uterine suture in the Cesarean operation is to keep the incised parts in close contact so as to prevent the escape of any fluid from the uterus into the peritoneal cavity, one should employ only such material as can be invariably relied upon.

In further proof of the position taken, Prof. Jenks quotes from the transactions of the Obstetrical Society of London, in which Drs. Routh, Meadows and others. give opinions unfavorable to the use of catgut for uterine sutures. Dr. Meadows mentions two cases of death after Cesarean section, attributable alone to the use of catgut for closing the urine rent. These, added the writer, are lessons which should be widely published. The operation having fallen into discredit on account of the great mortality which has resulted, the interests of humanity demand that everything which relates to its success or failure, should be brought to the ffnowledge of the medical public.—*Toledo Med. and Surg. Jour.*

